# Draft Genome Sequences of Two Carbapenemase-Producing *Acinetobacter baumannii* Clinical Strains Isolated from Albanian and Togolese Patients

**DOI:** 10.1128/genomeA.00115-17

**Published:** 2017-05-18

**Authors:** Mohamed M. H. Abdelbary, Guy Prod’hom, Gilbert Greub, Laurence Senn, Dominique S. Blanc

**Affiliations:** a Service of Hospital Preventive Medicine, Lausanne University Hospital, Lausanne, Switzerland; b Institute of Microbiology, Lausanne University Hospital, Lausanne, Switzerland

## Abstract

We report here the draft genome sequences of two multidrug-resistant *Acinetobacter baumannii* clinical strains, H31499 and H31506, which were isolated at the Lausanne University Hospital in 2015 from an Albanian and a Togolese patient, respectively.

## GENOME ANNOUNCEMENT

*Acinetobacter baumannii* is an important opportunistic pathogen that causes a wide range of nosocomial infections, including urinary tract, respiratory tract, and bloodstream infections (1, 2). In recent years, wide dissemination and hospital outbreaks of multidrug-resistant *A. baumannii* strains have been reported (3–5). Here, we report the draft genome sequences of two carbapenemase-producing *A. baumannii* strains, H31499 and H31506. Strain H31499 was isolated from an Albanian patient with infected burn wound, while strain H31506 was retrieved from a Togolese child with an infected surgical wound. Both of these patients were admitted at the Lausanne University Hospital in 2015.

The genomic DNA of both strains was extracted using the Wizard genomic DNA purification kit (Promega, Madison, WI, USA), according to the manufacturer’s instructions. Quantification of the extracted genomic DNA was performed using the Qubit double-stranded DNA high-sensitivity (HS) assay kit (Life Technologies, Inc., Waltham, MA, USA). Sequencing libraries were prepared using the Nextera XT DNA library preparation kit (Illumina, San Diego, CA, USA), according to the manufacturer’s guidelines, followed by sequencing using the version 2 chemistry protocol on the Illumina MiSeq platform (Illumina), generating 2 × 150-bp paired-end reads.

Multilocus sequence typing (MLST) and antimicrobial resistance gene profiling were performed *in silico* using SRST2 (6). Sequence reads were preprocessed and *de novo* assembled using the A5-miseq pipeline (7), with default parameters. Subsequently, annotations of the assembled draft genomes were performed using RAST (8).

The draft genome sequence of H31499 had 128 contigs that comprised 3,933,485 bp, with an *N*_*50*_ contig size of 125,943 bp and a G+C content of 38.8%. Similarly, the H31506 draft genome sequence consisted of 225 contigs, with an* N*_*50*_ contig size of 50,932 bp that made up a total length of 4,065,883 bp, with a G+C content of 39%.

*In silico* MLST analysis using the Pasteur Institute typing scheme (9) revealed that strains H31499 and H31506 belong to sequence type 2 (ST2) and ST575, respectively, while in the Oxford MLST scheme (10), they belong to ST436 and ST1051, respectively.

SRST2 analysis revealed that the genomes of strains H31499 and H31506 harbored 14 and 12 antimicrobial resistance genes, respectively. Genes that encode resistance to β-lactams (*bla*_Oxa-23_, *bla*_OXA-51_, *bla*_MBL_, and *ampC*), amikacin (*aphA6*), tetracyclines (*tetB*), and sulfonamides (*sul2*) were detected in both genomes. In addition, the H31498 genome harbored the genes *bla*_TEM-1_, *armA*, *aph(3′)-Ia*, *strA*, and *strB*, while the genes *bla*_CARB-2_, *bla*_PER-1_, *aadB*, and *sul1* were unique to the H31506 genome.

### Accession number(s).

The draft genome sequences of strains H31499 and H31506 have been deposited in the European Nucleotide Archive (ENA)/GenBank under BioProject PRJEB19200 and with accession numbers FTRW00000000 and FTRX00000000, respectively. The versions described in this paper are the first versions.
